# Promoting Ge Alloying Reaction via Heterostructure Engineering for High Efficient and Ultra‐Stable Sodium‐Ion Storage

**DOI:** 10.1002/advs.202002358

**Published:** 2020-10-08

**Authors:** Chaoqun Shang, Le Hu, Dan Luo, Krzysztof Kempa, Yongguang Zhang, Guofu Zhou, Xin Wang, Zhongwei Chen

**Affiliations:** ^1^ National Center for International Research on Green Optoelectronics South China Academy of Advanced Optoelectronics South China Normal University Guangzhou 510006 China; ^2^ Department of Chemical Engineering University of Waterloo Waterloo Ontario N2L 3G1 Canada; ^3^ International Academy of Optoelectronics at Zhaoqing South China Normal University Zhaoqing 526060 China; ^4^ Department of Physics Boston College Chestnut Hill MA 02467 USA

**Keywords:** Cu_3_Ge/Ge heterostructures, cycling stability, Germanium, sodium‐ion batteries, structural integrity

## Abstract

Germanium (Ge)‐based materials have been considered as potential anode materials for sodium‐ion batteries owing to their high theoretical specific capacity. However, the poor conductivity and Na^+^ diffusivity of Ge‐based materials result in retardant ion/electron transportation and insufficient sodium storage efficiency, leading to sluggish reaction kinetics. To intrinsically maximize the sodium storage capability of Ge, the nitrogen doped carbon‐coated Cu_3_Ge/Ge heterostructure material (Cu_3_Ge/Ge@N‐C) is developed for enhanced sodium storage. The pod‐like structure of Cu_3_Ge/Ge@N‐C exposes numerous active surface to shorten ion transportation pathway while the uniform encapsulation of carbon shell improves the electron transportation, leading to enhanced reaction kinetics. Theoretical calculation reveals that Cu_3_Ge/Ge heterostructure can offer decent electron conduction and lower the Na^+^ diffusion barrier, which further promotes Ge alloying reaction and improves its sodium storage capability close to its theoretical value. In addition, the uniform encapsulation of nitrogen‐doped carbon on Cu_3_Ge/Ge heterostructure material efficiently alleviates its volume expansion and prevents its decomposition, further ensuring its structural integrity upon cycling. Attributed to these unique superiorities, the as‐prepared Cu_3_Ge/Ge@N‐C electrode demonstrates admirable discharge capacity, outstanding rate capability and prolonged cycle lifespan (178 mAh g^−1^ at 4.0 A g^−1^ after 4000 cycles).

## Graphical abstract

Nitrogen doped carbon‐coated Cu_3_Ge/Ge heterostructure is developed as anode material for fast and longevous sodium storage. Cu_3_Ge/Ge heterostructure efficiently promotes the Na^+^ diffusion and electron conduction in the composite, which enhances the sodium storage capability of Ge and accelerates its redox reaction kinetics. Attributed to the structural superiorities, a remarkable discharge capacity, superb rate capability and enhanced cyclic stability can be realized.

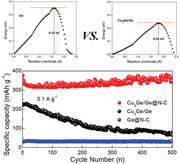

Sodium‐ion batteries (SIBs) have attracted great attention as one of the most promising energy storage devices.^[^ ^1^, ^2^, ^3^, ^4^ ^]^ However, the sluggish kinetics of Na^+^ diffusion results in undesirable electrochemical performance because of the large Na^+^ radius.^[^ ^5^, ^6^, ^7^ ^]^ Various anode materials have been investigated to achieve the reversible Na^+^ insertion/extraction, such as carbonaceous materials (hard carbons, expanded graphite, and graphene), alloy‐based materials, and metal oxides/sulfides.^[^ ^8^, ^9^, ^10^ ^]^ Unfortunately, graphite, the widely used anode material in commercial lithium‐ion batteries (LIBs), only delivers a low specific capacity of 50 mAh g^−1^ in traditional carbonate‐based electrolyte.^[^ ^11^, ^12^ ^]^ The exploration of appropriate anode materials for SIBs with high performance is challenging.^[^ ^13^, ^14^, ^15^ ^]^

Alloy‐type materials are promising candidates due to their large sodium storage capacity based on alloy/de‐alloy storage mechanism.^[^ ^16^, ^17^, ^18^, ^19^ ^]^ Among various alloy‐type materials, Ge has been found to electrochemically react with Na to form Na*_x_*Ge (369 mAh g^−1^) with an atomic ratio of about 1:1.^[^ ^20^, ^21^ ^]^ However, bulk Ge is undesirable for sodium storage because of the sluggish kinetics.^[^ ^22^ ^]^ Besides, the alloying reactions are usually accompanied by huge volume expansion of the host materials, which leads to anode degradation and poor capacity retention.^[^ ^23^, ^24^ ^]^ An effective strategy to enhance the reaction kinetics is to design nanostructured heterojunction, which cannot only short the sodium ion diffusion path, but also generate a built‐in electric field at the heterointerfaces, further improving the electrochemical reaction kinetics.^[^ ^25^, ^26^, ^27^ ^]^ Accompanied surface engineering such as carbon coating of nanosized active materials is commonly employed to prevent their pulverization and aggregation during cycling.^[^ ^28^, ^29^, ^30^, ^31^ ^]^ Thus, the combination of the two above‐mentioned tactics might realize high sodium storage performance of Ge with high rate capability and long‐term cycling stability.

In this work, a pod‐like Cu_3_Ge/Ge@N‐C is synthesized as anode material to realize the sodium storage of Ge. The design of Cu_3_Ge/Ge heterostructure can lower the Na^+^ diffusion barrier and facilitates electron transportation, rendering enhanced reaction kinetics and improved sodium storage capability of Ge. Meanwhile, the N‐C shell accommodates the volume expansion of Ge during alloying/dealloying process, which endows enhanced architecture integrity. Owing to these structural advantages, the optimal Cu_3_Ge/Ge@N‐C displays outstanding rate capability and long cycling stability with enhanced sodium storage property.

As shown in **Figure** **1**a, the Cu_3_Ge/Ge@N‐C was prepared by the in situ reduction of CuGeO_3_ nanowires with the aid of polydopamine (PDA). The CuGeO_3_ nanowire was initially coated by a uniform layer of PDA (CuGeO_3_@PDA). Subsequently, the CuGeO_3_@PDA nanorods undergone carbonization and in situ reduction leads to the formation of Cu_3_Ge/Ge@N‐C nanorods with pod‐like nanostructure. The presence of Cu_3_Ge/Ge heterostructure can intrinsically enhance the sodium storage efficiency of Ge. As displayed in Figure 1b, the as‐prepared CuGeO_3_ nanowires are dispersed regularly and exhibit well‐defined homogeneity. With coating of the PDA layer, the CuGeO_3_@PDA composite inherits the nanorod morphology without particle aggregation (Figure 1c). The TEM results in Figure 1e further demonstrate the nanowire structure of CuGeO_3_. The thickness of PDA coating is ≈9 nm, while the thickness of the inner CuGeO_3_ is ≈22 nm (Figure 1f), indicating the homogeneous coating. After reduction via pyrolysis, the CuGeO_3_ nanorods transformed into heterostructured Cu_3_Ge/Ge, which is wrapped in PDA‐derived nitrogen doped carbon layer, affording the formation of pod‐like Cu_3_Ge/Ge@N‐C composites (Figure 1d). As shown in Figure 1g, the Cu_3_Ge/Ge@N‐C exhibits a pod‐like nanostructure with admirable structure integrity, which is able to alleviate the volume variation during cycling. The high‐resolution TEM (HRTEM) of Cu_3_Ge/Ge@N‐C (Figure 1h) indicates its good crystallinity with lattice fringes of 0.20, 0.21, and 0.326 nm, which can be ascribed to (020), ($\bar{1}$11) plane of Cu_3_Ge and (111) plane of Ge, respectively. The TEM image (Figure 1i) and corresponding EDS elemental mapping show homogeneous element distribution of Ge, N, and C. For comparison, Cu_3_Ge/Ge was prepared by direct annealing of CuGeO_3_ nanowires. Without carbon encapsulation, severe aggregation can be observed from the SEM and TEM images of Cu_3_Ge/Ge (Figure S1, Supporting Information). Furthermore, the Ge@N‐C also displayed irregular bulk particles (Figure S2, Supporting Information).

**Figure 1:**
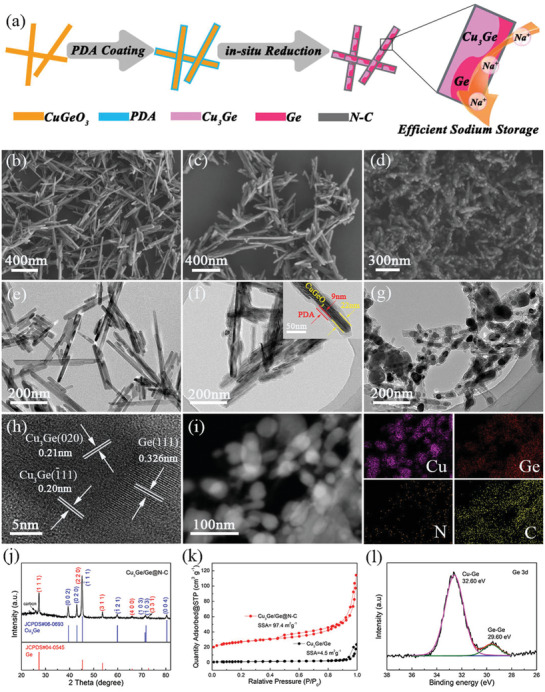
a) The schematic synthesis process of Cu_3_Ge/Ge@N‐C. b,e) SEM and TEM images of CuGeO_3_; c,f) CuGeO_3_@PDA; d,g) Cu_3_Ge/Ge@N‐C. h) HRTEM image of Cu_3_Ge/Ge@N‐C. i) The TEM and the corresponding EDS mapping of Cu_3_Ge/Ge@N‐C; j) XRD pattern of Cu_3_Ge/Ge@N‐C; k) N_2_ adsorption‐desorption isotherm Cu_3_Ge/Ge@N‐C and Cu_3_Ge/Ge; l) The high‐resolution XPS spectrum of Ge 3d of Cu_3_Ge/Ge@N‐C.

The XRD pattern of Cu_3_Ge/Ge@N‐C is shown in Figure 1j, where the diffraction peaks can be assigned to Cu_3_Ge (JCPDS#06‐0693) and Ge (JCPDS#04‐0545). The XRD patterns of Cu_3_Ge/Ge and Ge@N‐C are shown in Figure S4, Supporting Information, for comparison. The diffraction peaks are strong and sharp, indicating high crystallization.^[^ ^32^ ^]^ In contrast, Cu_3_Ge/Ge@N‐C shows broad diffraction peaks, confirming that the in situ formed Cu_3_Ge and nitrogen‐doped carbon prevent the aggregation of Cu_3_Ge/Ge particles. Moreover, the weak peak at ≈26 ° in the XRD pattern of Cu_3_Ge/Ge@N‐C carbon is attributed to the carbon coating. As clarified by the TGA, the carbon content in Cu_3_Ge/Ge@N‐C is ≈25.7 wt%, and the ratio of Ge in Cu_3_Ge/Ge@N‐C is ≈26.2% (Figure S4, Supporting Information). Figure 1k shows the N_2_ adsorption‐desorption isotherms of Cu_3_Ge/Ge@N‐C and Cu_3_Ge/Ge, respectively. The Cu_3_Ge/Ge@N‐C delivered a BET specific surface area of 97.4 m^2^ g^−1^, which is much higher than that of Cu_3_Ge/Ge (4.5 m^2^ g^−1^). Simultaneously, the mesoporous structure of Cu_3_Ge/Ge@N‐C shows a significantly higher pore volume of 0.18 cm^3^ g^−1^ (Figure S5a, Supporting Information). As shown in Figure S5b, Supporting Information, the Raman spectra of Cu_3_Ge/Ge@N‐C exhibits three peaks at 290 cm^−1^ (Ge), 1341 cm^−1^ (D band, disordered carbon) and at 1580 cm^−1^ (G band, graphene) with *I*_D_/*I*_G_ value of 0.85, respectively.^[^ ^33^ ^]^ The disordered carbon derived from N‐doping could enhance the electrochemical conductivity of carbon and further improve the sodium storage of Cu_3_Ge/Ge@N‐C. As clarified by XPS (Figure 1l), there are two peaks centered at 32.6 and 29.6 eV in Ge 3d spectrum, which are attributed to Cu‐Ge and Ge‐Ge, respectively.^[^ ^34^ ^]^ More XPS analysis details are shown in Figure S6, Supporting Information.

The electrochemical performance of Cu_3_Ge/Ge@N‐C is further evaluated. The initial three charge‐discharge cycles of Cu_3_Ge/Ge@N‐C are shown in **Figure** **2**a. In the first cycle, the Cu_3_Ge/Ge@N‐C shows high discharge capacity of 1170 mAh g^−1^ with a reversible charge capacity of 376 mAh g^−1^, which is mainly caused by solid electrolyte interface (SEI) formation and some irreversible side reactions, further confirmed by the CV results (Figure S7, Supporting Information).^[^ ^35^, ^36^ ^]^ The initial Coulombic efficiency of Cu_3_Ge/Ge@N‐C may be enhanced by decreasing the specific surface area to mitigate the side reactions or pre‐sodiation to offer extra sodium resource.^[^ ^37^, ^38^, ^39^ ^]^ In the subsequent cycles, the charge‐discharge curves are almost overlapped, indicating the high reaction reversibility. Furthermore, the ex situ (Figure S8, Supporting Information) XRD results reveal the sodium storage mechanism of Cu_3_Ge/Ge@N‐C: (i) the crystalline Ge converts into Na*_x_*Ge with low crystallinity after the initial discharge; (ii) after charge to 3.0 V, the amorphous Na_x_Ge transform to poor crystalline Ge with more active sites, thus ensuring favorable electrochemical performance for subsequent cycles. As depicted in Figure 2b and Figure S9, Supporting Information, Cu_3_Ge/Ge@N‐C delivers a high reversible capacity of 347 mAh g^−1^ after 500 cycles with high capacity retention of 99.3% and high Coulombic efficiency close to 100%, demonstrating high cycling stability. While the capacity of Cu_3_Ge/Ge electrode is only 65 mAh g^−1^ after 500 cycles, further revealing the important role of carbon coating to restrain structure degradation. The in situ formed Cu_3_Ge/Ge heterostructure is also the key factor to maximize the sodium storage efficiency of Ge. Without Cu_3_Ge, the Ge@N‐C displayed a specific capacity of only 40 mAh g^−1^, due to the sluggish reaction kinetics. The favorable electrochemical performance of Cu_3_Ge/Ge@N‐C could be derived from its well‐designed structure, which accommodates volumetric change by carbon coating during cycling, and achieves high Ge utilization efficiency via an in situ formed Cu_3_Ge/Ge heterostructure.

**Figure 2:**
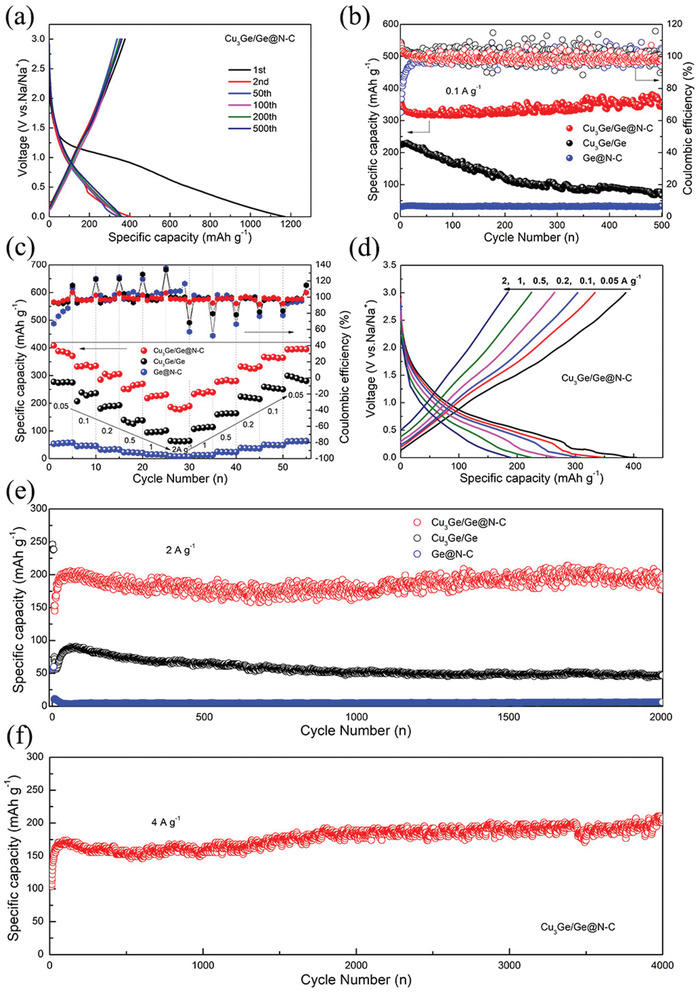
a) Charge‐discharge curves of Cu_3_Ge/Ge@N‐C; b) Cycling, c) rate, and e) long‐term cycling performance of the Cu_3_Ge/Ge@N‐C, Cu_3_Ge/Ge, and Ge@N‐C; d) charge‐discharge curves of Cu_3_Ge/Ge@N‐C at different current densities; f) long‐term cycling performance of Cu_3_Ge/Ge@N‐C at 4 A g^−1^.

The rate performances are shown in Figure 2c. As the current density increases, the corresponding reversible capacities of the Cu_3_Ge/Ge@N‐C electrode decrease. A highly reversible specific capacity of 394 mAh g^−1^ is obtained when the current density gradually recovers to 0.05 A g^−1^, which is close to the initial value. However, without N‐C or Cu_3_Ge, Cu_3_Ge/Ge and Ge@N‐C show inferior specific capacity. Furthermore, the Cu_3_Ge/Ge@N‐C electrode in sodium storage exhibits promising electrochemical performance (Table S1, Supporting Information). The representative charge‐discharge curves of Cu_3_Ge/Ge@N‐C at different current densities are presented in Figure 2d, which exhibits a higher discharge capacity than that of Cu_3_Ge/Ge (Figure S10a, Supporting Information) and Ge@N‐C (Figure S10b, Supporting Information). Also, Cu_3_Ge/Ge@N‐C possessed considerable cycling stability at 2 A g^−1^ (see Figure 2e). For the initial 10 cycles, the specific capacity increases from 150 to 205 mAh g^−1^, indicating an electrochemical activation process.^[^ ^40^ ^]^ After 2000 cycles, the specific capacity of Cu_3_Ge/Ge@N‐C remains stable at 195 mAh g^−1^. Even at high current density of 4 A g^−1^, the Cu_3_Ge/Ge@N‐C keeps a reversible capacity of 178 mAh g^−1^ after 4000 cycles, (Figure 2f). It is undoubtedly that the exterior N‐C shell accommodates volume expansion and alleviates the stress of Cu_3_Ge/Ge, which prevents particle aggregation and collapse during Na^+^ alloying/dealloying, ensuring high cycling stability of Cu_3_Ge/Ge@N‐C.

Hence, to reveal the structural stability, the morphologies of Cu_3_Ge/Ge@N‐C before and after 100 GCD cycles are presented. The as‐prepared Cu_3_Ge/Ge@N‐C electrode is flat and smooth with regular pod‐like nanostructure (**Figure** **3**a,b). After 100 cycles, the Cu_3_Ge/Ge@N‐C still keeps the structural integrity, where the active materials tightly contact with the current collector (Figure 3c). Besides, the existence of SEI covers the surface of pod‐like active materials (Figure 3d). As shown in Figure S11, Supporting Information, without the encapsulation of N‐C, severe electrode pulverization and agglomeration of Cu_3_Ge/Ge can be observed, which induces material pilling off from the current collector during cycling and results in poor cycling stability. Furthermore, the charge transfer resistance (*R*_ct_) of Cu_3_Ge/Ge@N‐C is slightly lower than that of Cu_3_Ge/Ge (169 versus 223 Ω), indicating that the N‐C layer is beneficial to the charge transportation kinetics initially (Figure 3e). After 100 GCD cycles, the *R*_ct_ of Cu_3_Ge/Ge@N‐C increases to ≈355 Ω, which is smaller than that of Cu_3_Ge/Ge (519 Ω), further demonstrating that the N‐C layer could also contribute to the stable SEI formation and structural integrity (Figure 3f).

**Figure 3:**
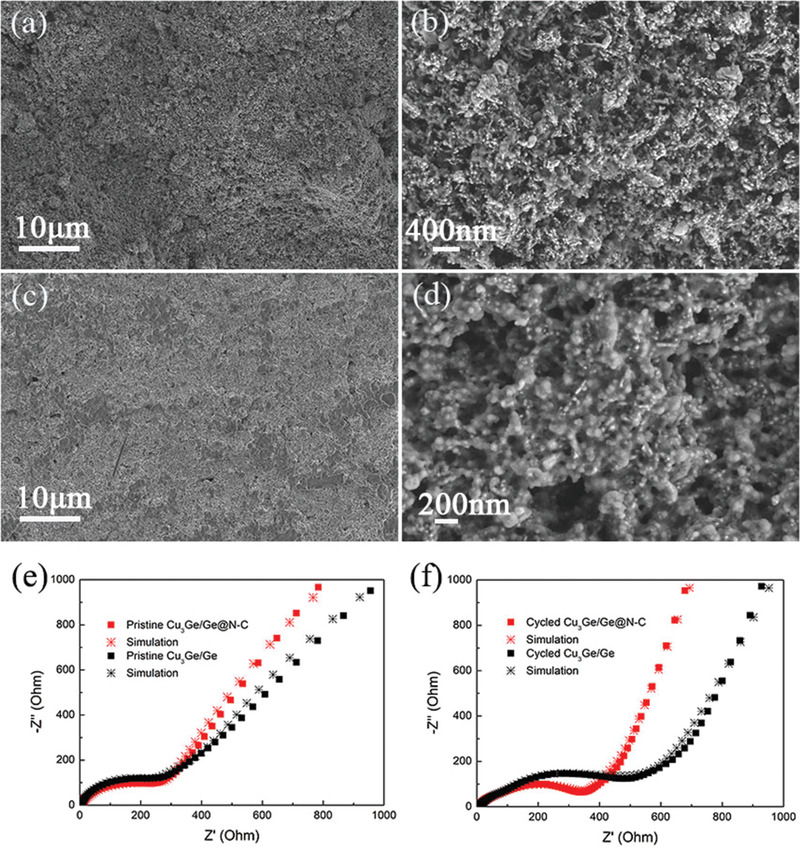
a,b) SEM images of Cu_3_Ge/Ge@N‐C electrode before and c,d) after 100 cycles at a current density of 0.1 A g^−1^. e) The EIS spectra and corresponding simulation of Cu_3_Ge/Ge@N‐C and Cu_3_Ge/Ge before and f) after cycling, respectively.

The Cu_3_Ge/Ge@N‐C displays remarkably enhanced rate capability and cycling performance, compared to those of Cu_3_Ge/Ge and Ge@N‐C, which could be attributed to the synergistic effect of the exterior N‐C coating layer, and the intrinsic Cu_3_Ge/Ge heterojunction. Most importantly, the intrinsic heterostructure of Cu_3_Ge/Ge improves fundamentally the sodium storage property of Ge. To clarify this, we have performed the DFT calculations. **Figure** **4**a shows the optimized state of Na atom on the Cu_3_Ge/Ge facet. The adsorption energy of Na atom on Cu_3_Ge/Ge is −2.53 eV, which is much lower than −0.58 eV of Ge (Figure S12a, Supporting Information), indicating that the Na atom is more easily absorbed on the Cu_3_Ge/Ge. And the diffusion barrier of Na atom on Cu_3_Ge/Ge (0.24 eV, Figure 4b) is lower than that on Ge (0.35 eV, Figure S12b, Supporting Information), demonstrating that Na^+^ diffusion is energetically favorable on the Cu_3_Ge/Ge. In the case of heterojunction structure derived from Cu_3_Ge/Ge, no bandgap could be found at the Fermi level (Figure 4c,d), while there is an obvious band gap existing in the bulk Ge (Figure S12c and d, Supporting Information). These DFT results suggest that the Cu_3_Ge/Ge heterostructure can enhance the electronic conductivity, thus contributing to the fast reaction kinetics to some extent.^[^ ^41^, ^42^ ^]^

**Figure 4:**
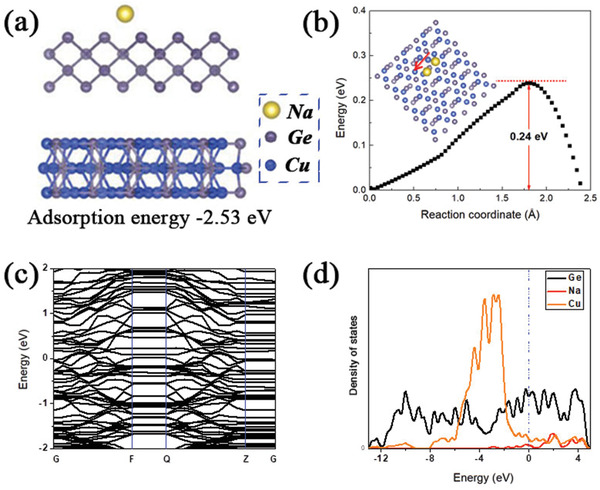
The optimized state of the diffusion of a) Na atom on Cu_3_Ge/Ge and b) corresponding diffusion barrier; c) the band structure and d) DOS of Na atom adsorbed on Cu_3_Ge/Ge.

The N‐C layer could effectively protect the Cu_3_Ge/Ge heterostructure from collapse and enhance the reaction kinetics, demonstrating improved electrochemical performance. To investigate the kinetics process of the Cu_3_Ge/Ge@N‐C and Cu_3_Ge/Ge, the CV tests at various scan rates from 0.2 to 1.0 mV s^−1^ are carried out as shown in **Figure** **5**a and Figure S13a, Supporting Information. The relationship between the sweep rate (v, mV s^−1^) and the peak current (i, mA) is described in the following equation:^[^ ^43^ ^]^

(1) $$\begin{array}{c} i=av^{b} \end{array}$$

(2) $$\begin{array}{c} \log\left(i\right)=\log\left(a\right)+b\log\left(v\right) \end{array}$$

where the *b* value of 0.5 or 1.0 represented diffusion‐controlled or capacitive processes, respectively.^[^ ^43^, ^44^ ^]^ From Figure 5b, the *b*‐value of Cu_3_Ge/Ge@N‐C electrode is calculated to be 0.89 and 0.94 for anodic peak and cathodic peak, indicating dominant capacitive processes. Figure S13b, Supporting Information, illustrates that the *b*‐value of Cu_3_Ge/Ge is 0.75 and 0.92, respectively. In addition, the total capacitive contribution can be divided into capacitive contribution (*k*_2_ *υ*) and diffusion‐controlled contribution (*k*_1_ *υ^1/2^*):^[^ ^45^ ^]^

(3) $$\begin{array}{c} i=k_{1}\upsilon^{1/2}+k_{2}\upsilon \end{array}$$

(4) $$\begin{array}{c} i/\upsilon^{1/2}=k_{1}+k_{2}\upsilon^{1/2} \end{array}$$

**Figure 5:**
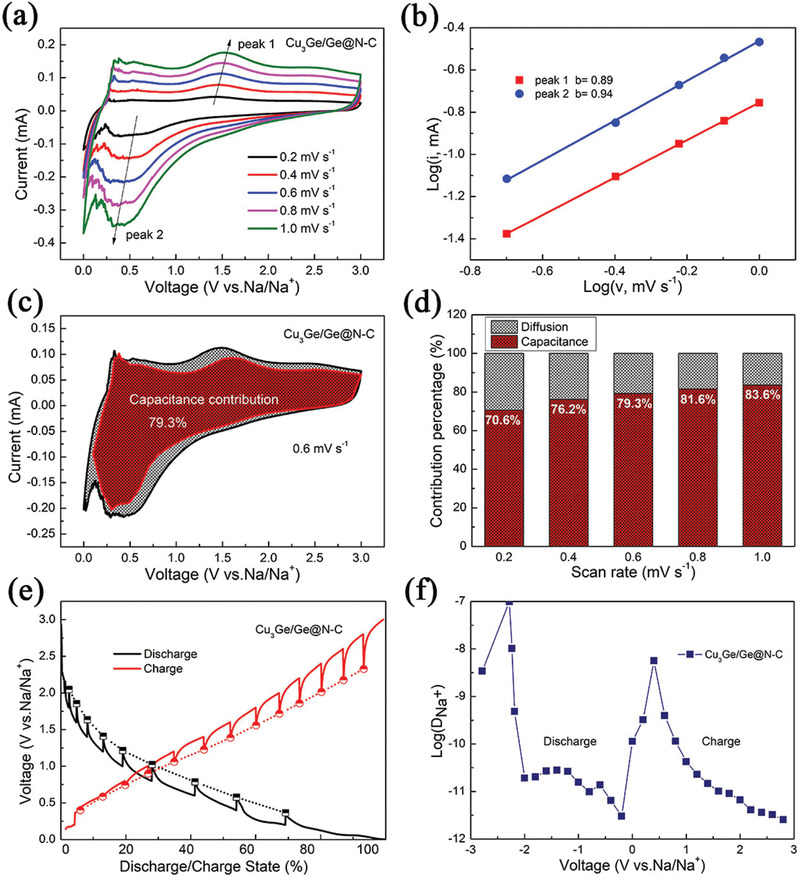
Cu_3_Ge/Ge@N‐C: a) CV curves at the scan rates from 0.2 to 1.0 mV s^−1^; b) b values according to log(*i*) and log(*υ*) of the oxidation peak and reduction peak; c) capacitive storage contributions to total measured current at 0.6 mV s^−1^; d) relative capacitive contribution at different scan rates; e) GITT curves of Cu_3_Ge/Ge@N‐C in discharge‐charge process; f) corresponding Na^+^ diffusion coefficients.

In Figure 5c, the capacitive‐controlled region is shaded in the total measured current at 0.6 mV s^−1^ for Cu_3_Ge/Ge@N‐C. The calculated capacitive contribution of Cu_3_Ge/Ge@N‐C is 79.3%, which is higher than that of Cu_3_Ge/Ge (Figure S13c, Supporting Information, 57.1%), suggesting that Cu_3_Ge/Ge@N‐C possesses favorable charge‐transfer kinetics. Figure 5d summarizes the capacitive‐controlled contributions of Cu_3_Ge/Ge@N‐C at scan rates of 0.2, 0.4, 0.6, 0.8, and 1.0 mV s^−1^, corresponding to the capacitive contribution of 70.6%, 76.2%, 79.3%, 81.6%, and 83.6%, higher than those of Cu_3_Ge/Ge (Figure S13d, Supporting Information). Cu_3_Ge/Ge@N‐C electrode displays dominant capacitive charge storage under high current density, which is in accordance with the promising rate capability (Figure 3c).

Furthermore, the galvanostatic intermittent titration technique (GITT) is introduced to investigate the Na^+^ diffusion coefficient (*D*_Na_^+^). A current pulse is applied for 10 min with a subsequent relaxing time for 3 h to reach equilibrium potential state. Figure 5e and Figure S14, Supporting Information, shows the GITT curves of Cu_3_Ge/Ge@N‐C and Cu_3_Ge/Ge. The apparent *D*_Na+_ can be calculated according to the following equation:^[^ ^46^ ^]^

(5) $$\begin{array}{c} D_{\mathrm{Na}+}=\frac{4}{\pi \tau }{\left(\frac{mV_{m}}{MA}\right)}^{2}{\left(\frac{\Delta E_{s}}{\Delta E_{\tau }}\right)}^{2} \end{array}$$

where *τ* is the current pulse time (s), *m* and *M* are the mass and molar mass of Ge, and *V_m_* is the molar volume of the active materials, *A* is the surface area of the electrode. Δ*E_s_* and Δ*E_*τ*_* are potential variations of quasi‐equilibrium potential and potential variation during a constant current pulse.^[^ ^47^ ^]^ Accordingly, the calculated *D*_Na_^+^ of Cu_3_Ge/Ge@N‐C and Cu_3_Ge/Ge in the first discharge‐charge process is presented in Figure 5f and Figure S14, Supporting Information, respectively. The *D*_Na_^+^ of Cu_3_Ge/Ge@N‐C is in the range of 10^−12^ to 10^−10^ cm^2^ s^−1^ comparable to that of Cu_3_Ge/Ge.^[^ ^46^ ^]^ This reveals that the N‐C layer has a slight effect to the ionic conductivity. The exterior N‐C layer is aimed to keep the structural stability and enhance electrical conductivity. In combination with interior Cu_3_Ge/Ge heterostructure construction, the Cu_3_Ge/Ge@N‐C shows promising sodium storage capability with comparable cycling performance and rate capability.

In conclusion, we have successfully fabricated the Cu_3_Ge/Ge@N‐C composite electrode with a unique pod‐like nanostructure, with a great potential for applications in SIBs. The sodium storage capability of Ge is greatly enhanced because of the formation of Cu_3_Ge/Ge heterostructure, which can effectively lower the Na^+^ diffusion barrier and facilitates ion/electron transportation, leading to improved Na^+^ reaction kinetics. The uniform carbon encapsulation also alleviates the volume expansion during alloying/dealloying process, offering enhanced structure integrity. When employed Cu_3_Ge/Ge@N‐C as anode material for SIBs, it demonstrates excellent rate capability (385 mAh g^−1^ at 0.05 A g^−1^; 187 mAh g^−1^ at 2.0 A g^−1^), and long cycling stability (178 mAh g^−1^ at 4.0 A g^−1^ after 4000 cycles), holding great promises to be used for practical application.

## Experimental Section

##### Synthesis of CuGeO_3_ Nanowires

The CuGeO_3_ nanowires were obtained via a simple hydrothermal method. 0.1 g cetyltrimethylammonium bromide (CTAB) was dissolved in 15 mL of distilled water (DIW) to form a homogeneous suspension under continuously stirring for 1 h. Next, 5 mm Cu(CH_3_COO)_2_·H_2_O and 5 mm GeO_2_ were added to the above solution, respectively, and the mixed solution was stirred continuously for 1 h. Afterward, the above solution was transferred into a 20 mL Teflon‐lined stainless steel autoclave and then kept at 180 °C for 24 h. Finally, the blue products were collected by centrifugation and washed with deionized ethanol and water several times and dried via freeze‐drying at −40 °C for 24 h.

##### Synthesis of Cu_3_Ge/Ge@N‐C, Cu_3_Ge/Ge, and Ge@N‐C

125 mg of CuGeO_3_ nanowires were dispersed in a 50 mL of Trisbuffer (10 × 10^−3^ m). Then, 50 mg of dopamine hydrochloride was added to the mixture. After stirring for 10 h, the CuGeO_3_@PDA was collected by centrifugal separation, washed with deionized water, and dried via freeze‐drying at −40 °C for 24 h. Finally, Cu_3_Ge/Ge@N‐C was prepared through calcination treatment in 10% Ar/H_2_ atmosphere at 650 °C for 2 h with a heating rate of 2 °C min^−1^. Cu_3_Ge/Ge was prepared via a direct annealing CuGeO_3_ without PDA coating in 10% Ar/H_2_ atmosphere at 650 °C for 4 h with a heating rate of 2 °C min^−1^. Ge@N‐C was prepared through two main processes. First, Ge nanoparticle was obtained by reducing carboxyethylgermanium sesquioxide (Ge132) in 10% Ar/H_2_ atmosphere at 700 °C for 2 h. Then, Ge@N‐C was collected by stirring Ge and PDA with a molar 2.5:1 ratio and annealing in 10% Ar/H_2_ atmosphere at 650 °C for 2 h.

## Conflict of Interest

The authors declare no conflict of interest.

## Supporting information

Supporting Information
