# Supplementary material for: Promoting Ge Alloying Reaction via Heterostructure Engineering for High Efficient and Ultra‐Stable Sodium‐Ion Storage
Source: Adv Sci (Weinh). 2020 Oct 8;7(22):2002358. doi: 10.1002/advs.202002358 (PMC7675052; doi:10.1002/advs.202002358)
Supplement: Supplementary file 1 — Supporting Information [file ADVS-7-2002358-s001.pdf]

© 2020 Wiley-VCH GmbH

ADVANCED SCIENCE
Open Access

Supporting Information

for *Adv. Sci.,* DOI: 10.1002/advs.202002358

Promoting Ge Alloying Reaction via Heterostructure Engineering for High efficient and Ultra-stable Sodium-ion Storage

Chaoqun Shang, Le Hu, Dan Luo, Krzysztof Kempa, Yongguang Zhang, Guofu Zhou, Xin Wang, Zhongwei Chen**

Supporting Information

Promoting Ge Alloying Reaction via Heterostructure Engineering for High efficient and Ultra-stable Sodium-ion Storage

Chaoqun Shang, Le Hu, Dan Luo, Krzysztof Kempa, Yongguang Zhang, Guofu Zhou, Xin Wang, Zhongwei Chen**

Materials Characterization

The morphology of the samples were characterized by SEM (FESEM; ZEISS Ultra 55) and TEM (JEOL JEM-2100F). X-ray diffraction (XRD) patterns were collected by using Bruker-AXS Micro-diffractometer (D8 ADVANCE) under CuKα radiation (λ = 1.5406 Å) at a voltage of 30 kV. Thermogravimetric analysis (TGA) was performed using a Mettler−Toledo Model TGA/DSC1 system in air using a heating rate of 10 °C min^{-1}. The specific surface areas were calculated using the Barrett−Emmett−Teller (BET) method with a porosimeter (ASAP 2020, Micrometrics, Inc.). The pore size distribution was analyzed using desorption branch of the isotherms by the Barrett-Joyner-Halenda model. XPS analysis was obtained by a Thermo Fisher ESCALAB 205Xi.

Electrochemical Measurements

The electrochemical performances of the anodes were tested in CR2032 coin cells, which were assembled in an Ar-filled glove box. A slurry of active material, Super P, PVDF (80, 10, and 10 wt%) and N-methyl-2-pyrrolidone was spread onto Cu foil current collectors and then dried under vacuum at 70℃ for 12 h. The electrode film was punched into the discs with a diameter of 12 mm and dried at 60 °C for 24 h under vacuum oven. The mass loading of active material is 0.8-1.2 mg cm^{-2}.The electrolyte was 1 M $NaClO_4$ salt dissolved in ethylene carbonate/dimethyl carbonate (EC/DMC, 1:1 v/v) with 5 vol% fluoroethylene carbonate (FEC) as additive. Sodium foil was used as the counter electrode, and glass microfiber (Whatman) as the separator. Cycling and rate performance were tested on a NEWARE measurement system. CV analysis were conducted using the CHI660E electrochemical work station with a voltage range of 0.001-3.0 V (vs Na/Na^+) at various sweep rates. And the specific capacity was calculated based on the active Ge component.

DFT calculations

All the geometry optimization in this work were performed with the VASP code[1], using the GGA approximation in the form of the Perdew-Burke-Ernzerhof (PBE)[2] exchange-correlation functional. The projected augmented wave method (PAW)[3]

was used to describe the interaction between the valence electrons and the core. The adsorption energy ΔE_{ad} was calculated using:

$$\Delta E_{ad} = E(X + Na) - E_X - E_{Na}$$

where $E(X + Na)$ stand for the total energy of Ge or Cu_3Ge@Ge with 1 Na atom adsorbed on the facet, E_X is the total energy of Ge or Cu_3Ge@Ge, E_{Na} is the energy of Na atom.

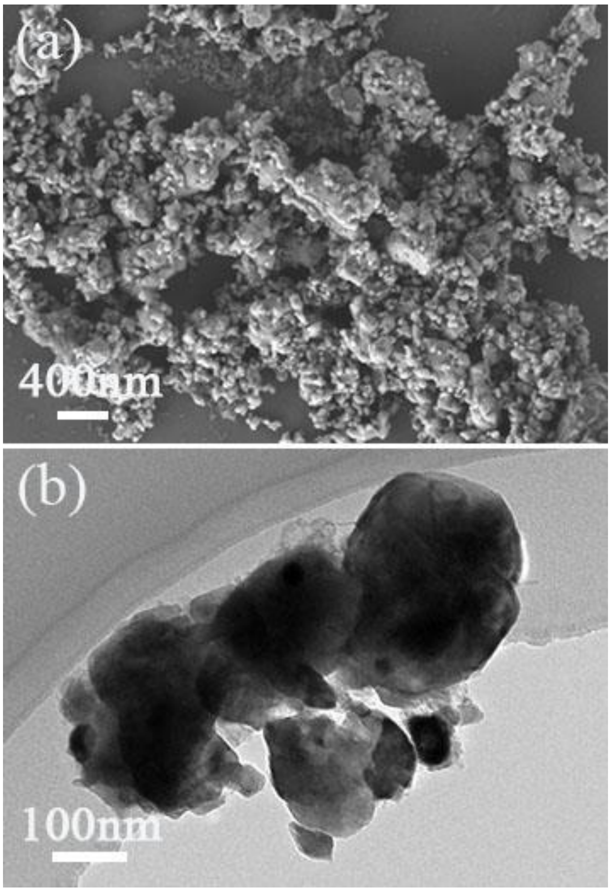

Figure S1. Typical (a) SEM and (b) TEM images of Cu_3Ge/Ge.

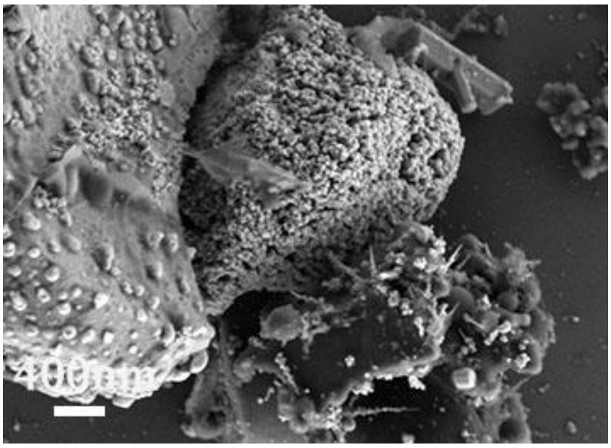

Figure S2. Typical SEM image of Ge@N-C.

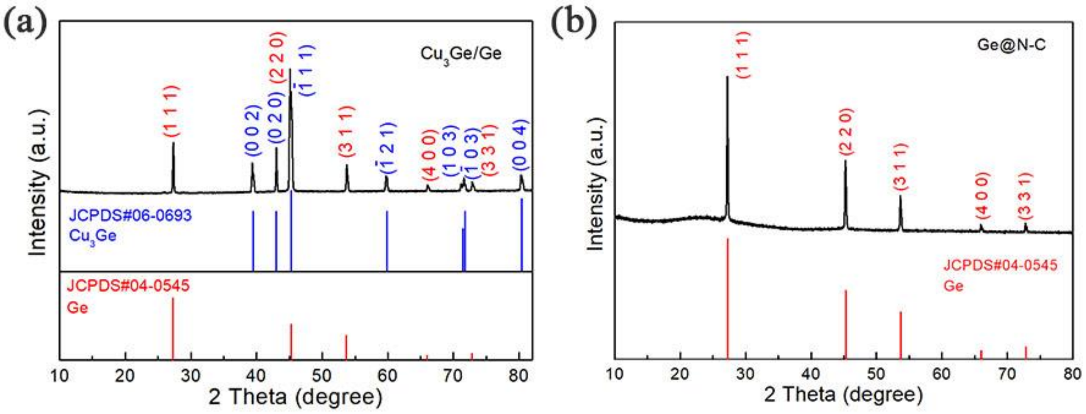

Figure S3. XRD patterns of (a) Cu_3Ge/Ge and (b) Ge@N-C, respectively.

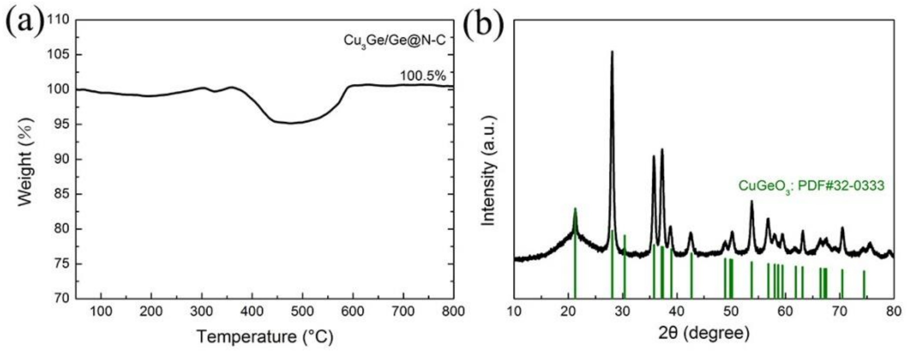

Figure S4. (a) TGA pattern of $Cu_3Ge/Ge@N\text{-}C$; (b) XRD pattern of the combustion product of $Cu_3Ge/Ge@N\text{-}C$.

The TGA pattern of $Cu_3Ge/Ge@N\text{-}C$ is shown in Figure S4a. As the temperature increases, N-C in $Cu_3Ge/Ge@N\text{-}C$ is burning up accompanied by the oxidation of Cu_3Ge/Ge. The final product of $Cu_3Ge/Ge@N\text{-}C$ after the same procedure of TGA is $CuGeO_3$ (Figure S4b). The corresponding carbon content in $Cu_3Ge/Ge@N\text{-}C$ is based on the law of conservation of mass. And the calculation details are as follows:

For the combustion product of $CuGeO_3$:

$$\mathrm{W(Cu + Ge)} = \frac{\mathrm{M(Cu)+M(Ge)}}{\mathrm{M(Cu)+M(Ge)+M(O)}} * 100.5\%$$

$$= \frac{63.5+72.6}{63.5+72.6+16*3} * 100.5\%$$

$$= 74.3\%$$

For the initial material of $Cu_3Ge/Ge@N\text{-}C$:

$$\mathrm{W(N - C)} = 100\% - \mathrm{W(Cu + Ge)}$$

$$= 100\% - 74.3\%$$

$$= 25.7\%$$

For the ratio of Ge in $Cu_3Ge/Ge@N\text{-}C$:

$CuGeO_3$ → $Cu_3Ge + 2Ge$

$\mathrm{W(Cu + Ge)}$= 74.3%

$$\text{Ge wt\%} = \frac{2M(Ge)}{M(Cu3Ge)+2M(Ge)} * 74.3\%$$

$$= \frac{2*72.6}{63.5*3+72.6+2*72.6}*74.3\%$$

$$= 26.2\%$$

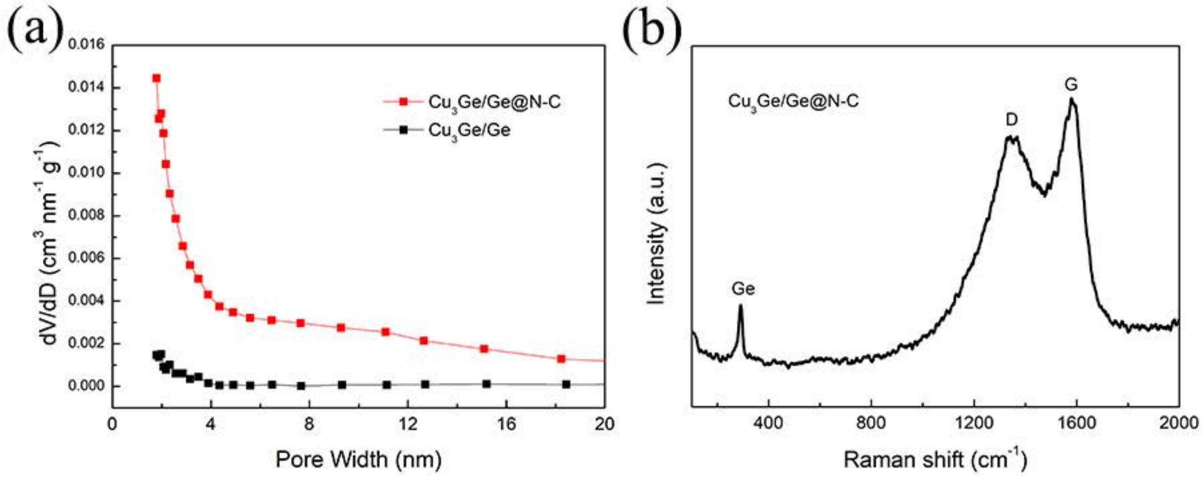

Figure S5. (a) The pore size distribution of $Cu_3Ge/Ge@N$-C and Cu_3Ge/Ge. (b) Raman spectra of $Cu_3Ge/Ge@N$-C.

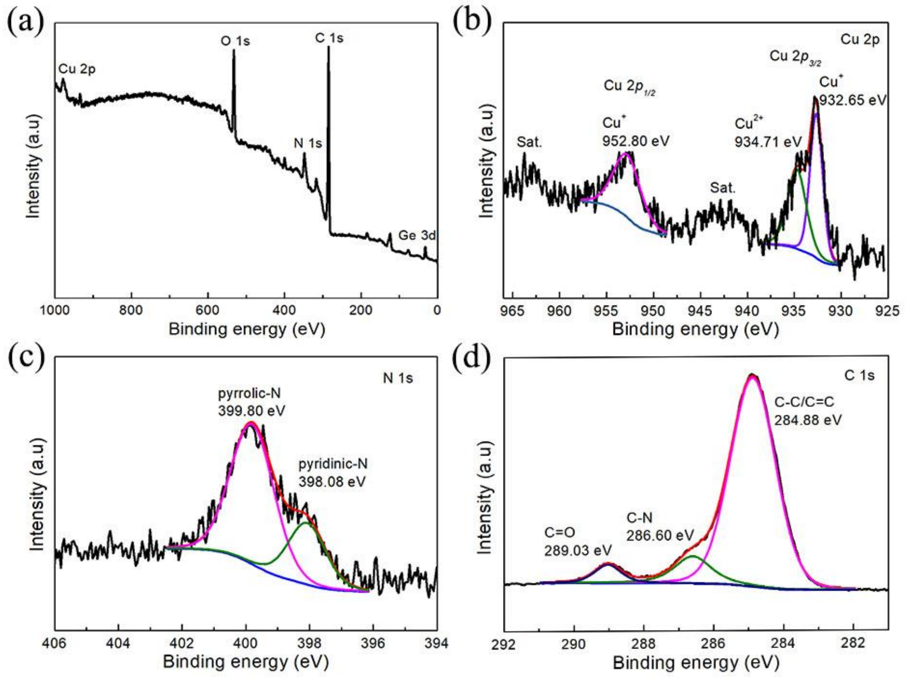

Figure S6. The survey spectrum (a) and high-resolution XPS spectra of (b) Cu 2p, (c) N 1s and (d) C 1s of the Cu_3Ge/Ge@N-C, respectively.

Figure S6a displays the survey spectrum of Cu_3Ge/Ge@N-C. The Cu 2p spectrum (Figure S6b) is specified as Cu $2p_{3/2}$ and Cu $2p_{1/2}$ due to the multiplet splitting. The Cu $2p_{3/2}$ peak is split into two pairs of peaks related to Cu^+ (932.65 eV) and Cu^{2+} (934.71 eV) valences. The Cu $2p_{1/2}$ peaks at 952.80 eV belong to Cu^+ species. The N 1s spectrum (Figure S6c) can be deconvoluted into two peaks that are ascribed to pyridinic-N (398.08 eV), pyrrolic-N (399.80 eV). In the C 1s spectrum of Figure S6d, there are three peaks located at 284.88, 286.60, and 289.03 eV, which are ascribed to C-C/C=C, C−N, and C=C, respectively.

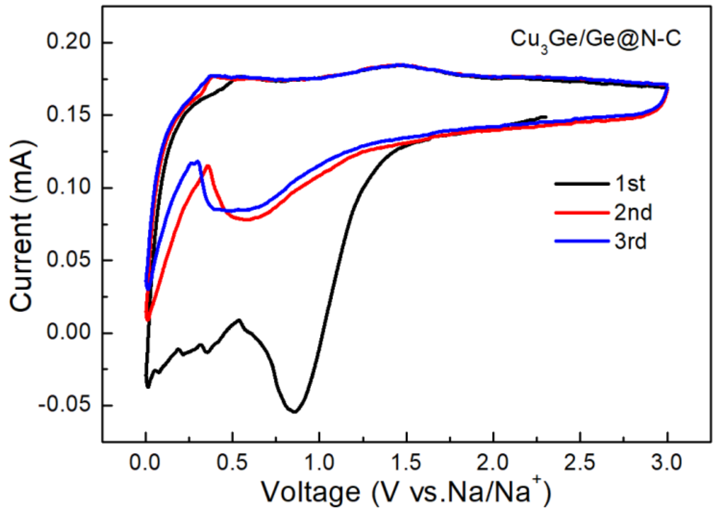

Figure S7. CV curves of the initial three cycles for the Cu_3Ge/Ge@N-C electrode at a scan rate of 0.2 mV s^{-1}.

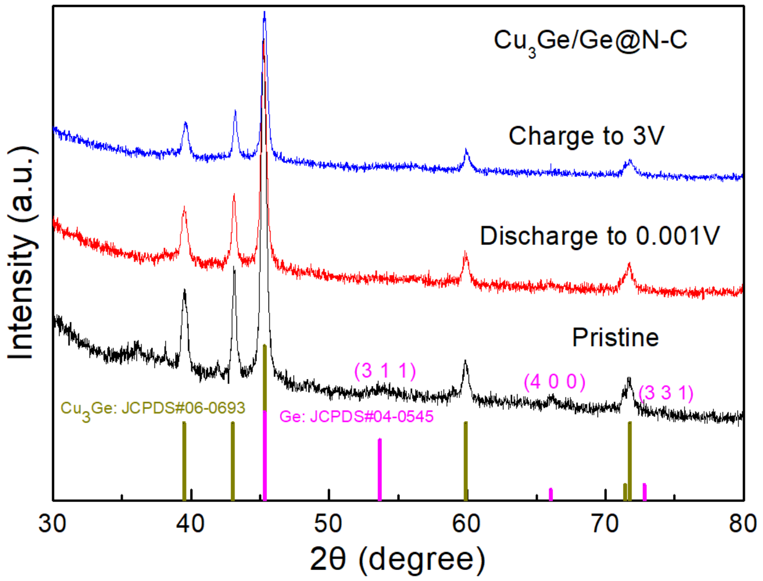

Figure S8. Ex situ XRD patterns of the Cu_3Ge/Ge@N-C electrode under different state: pristine, discharge to 0.001 V, and charge to 3 V, respectively.

As shown in the ex-situ XRD patterns (Figure S8), the (311), (400) and (311) peaks of Ge can be detected in the pristine Cu_3Ge/Ge@N-C electrode. When discharged to 0.001 V, the XRD signals of Ge disappear while the diffraction of peaks Cu_3Ge show no change, indicating the alloying reaction between sodium ion and Ge. When charged to 3 V, the XRD pattern displays similar signals to that of discharged state probably owing to the poor crystallinity of charged and discharged products.

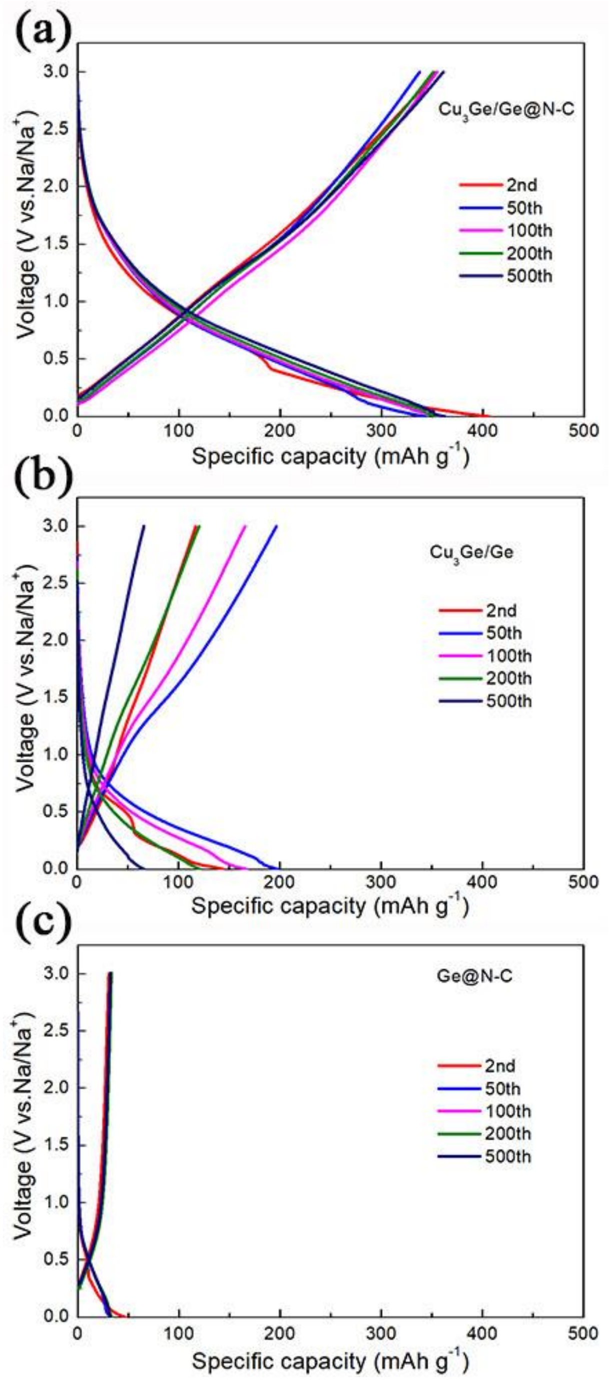

Figure S9. Charge-discharge curves of $Cu_3Ge/Ge@NC$, Cu_3Ge/Ge and Ge@N-C, respectively.

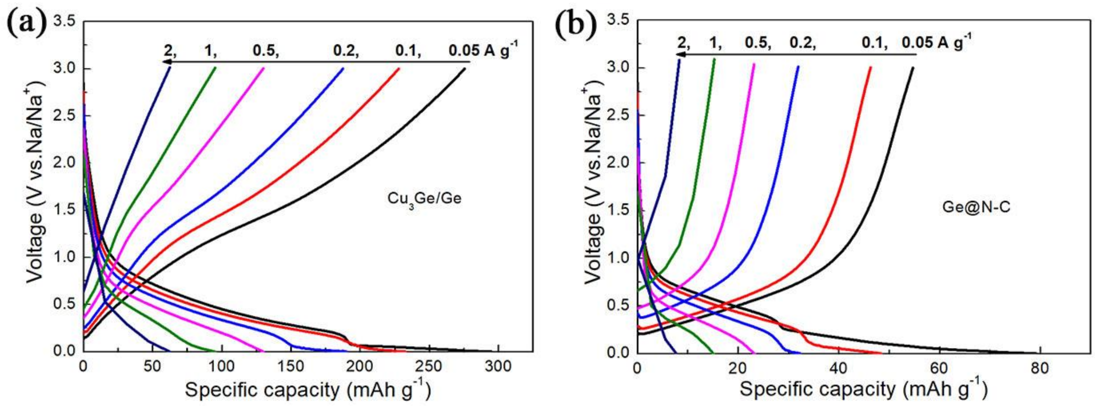

Figure S10. Charge-discharge curves of (a) Cu_3Ge/Ge and (b) Ge@N-C at different current densities.

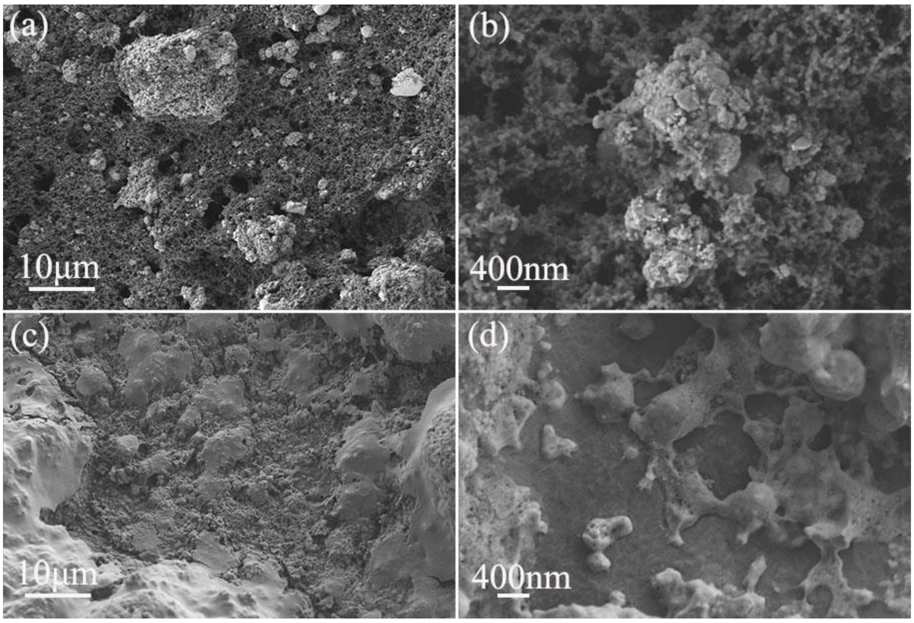

Figure S11. (a,b) SEM images of Cu_3Ge/Ge electrode before cycling. (c,d) SEM images of Cu_3Ge/Ge electrode after 100 cycles at a current density of 0.1 A g^{-1}.

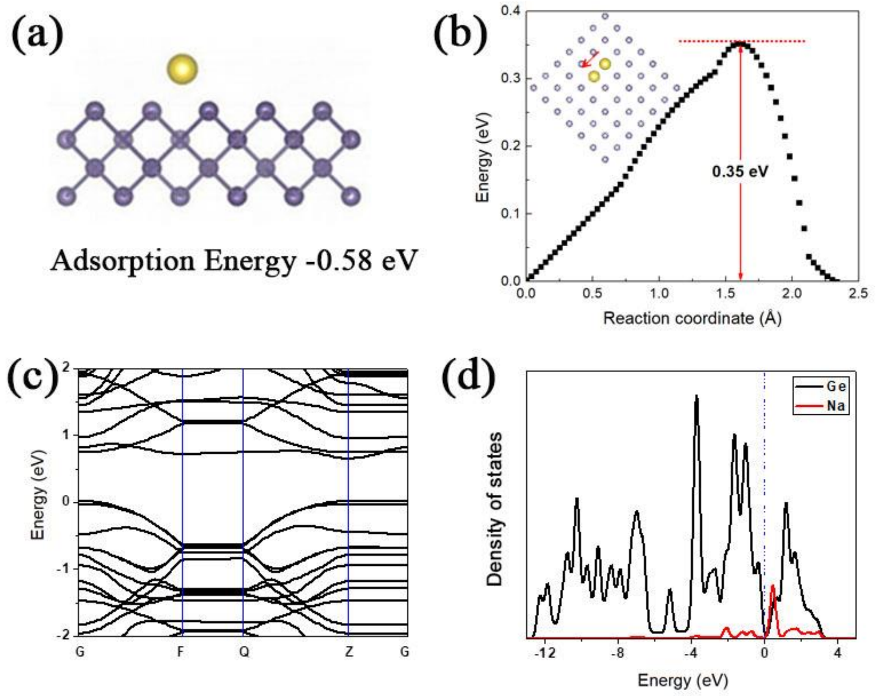

Figure S12. The optimized state of the diffusion of (a) Na atom on Ge and (b) corresponding diffusion barrier; (c) the band structure and (d) DOS of Na atom adsorbed on Ge.

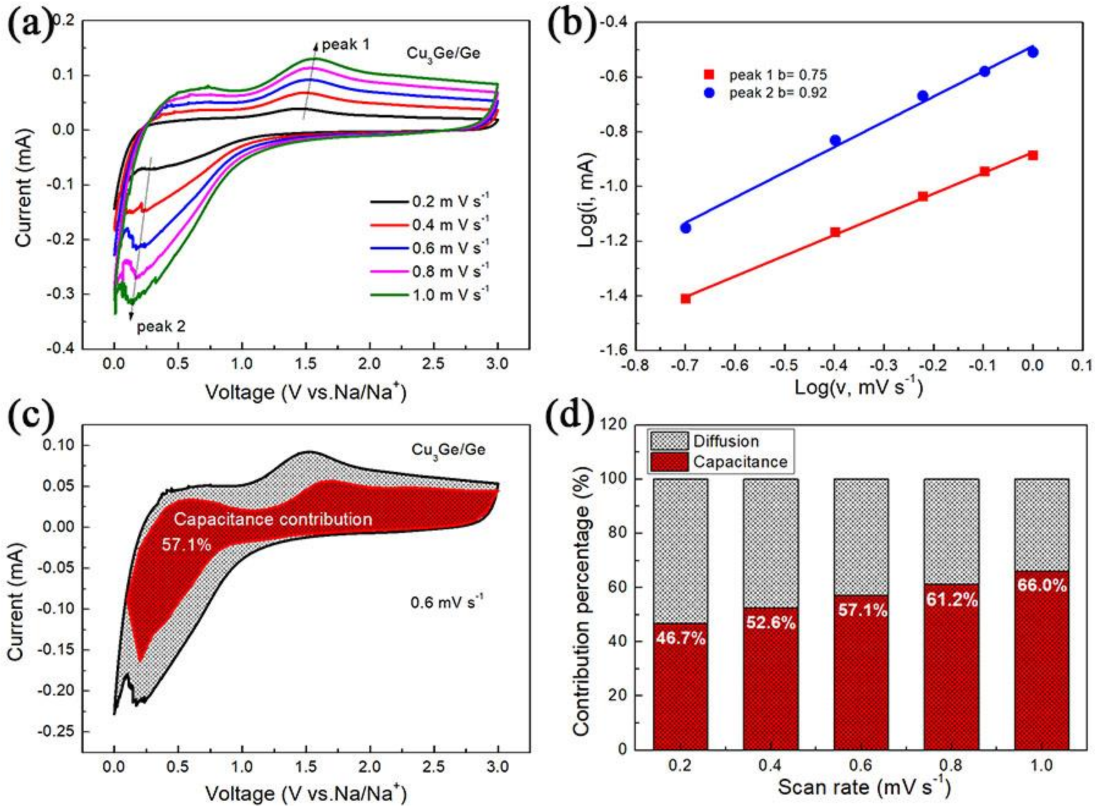

Figure S13. Cu_3Ge/Ge: (a) CV curves at the scan rates from 0.2 to 1.0 mV s^{-1}; (b) b values according to log(i) and log(υ) of the oxidation peak and reduction peak; (c) capacitive storage contributions to total measured current at 0.6 mV s^{-1}; (d) relative capacitive contribution at different scan rates.

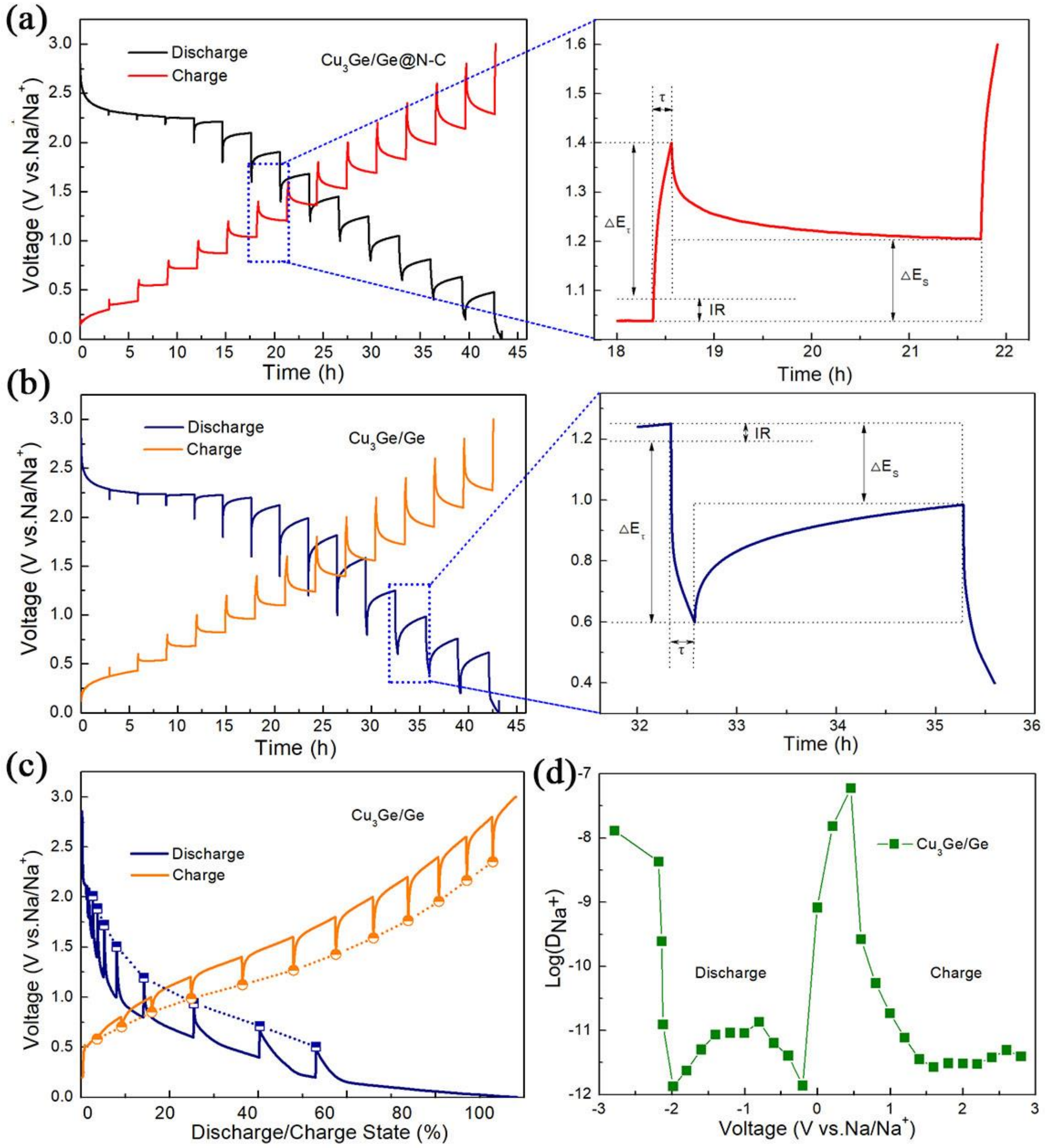

Figure S14. GITT curves of (a) Cu_3Ge/Ge@N-C and (b) Cu_3Ge/Ge; (c) GITT analysis of Cu_3Ge/Ge in discharge-charge process; (d) corresponding Na^+ diffusion coefficients.

Table S1. A comparison of the electrochemical performances of $Cu_3Ge/Ge@N\text{-}C$ (this work) and other Ge-based electrodes in sodium storage.

Samples	Sodium storage	
	Cycling performance (reversible capacity)	Rate performance
Cu_3Ge/Ge@N-C (This work)	347 mAh g^{-1} after 500 cycles (0.1 A g^{-1})	201 mAh g^{-1} at 2 A g^{-1}
Ge@G@TiO_2 Nanofibers (Adv. Funct. Mater. 2016, 26, 1104-1111.)	190 mAh g^{-1} after 100 cycles (0.1 A g^{-1})	88 mAh g^{-1} at 1 A g^{-1},
hollow Ge/C hybrids (Part. Part. Syst. Char. 2017, 34, 1600115.)	346 mAh g^{-1} after 500 cycles (0.1 A g^{-1})	112 mAh g^{-1} at 1 A g^{-1}
Ge-NMCFs (Nanotechnology 2020, 31, 015402.)	160 mAh g^{-1} after 700 cycles (0.5 A g^{-1})	153 mAh g^{-1} at 0.74 A g^{-1} (2 C)

Refenences

[1] G. Kresse, J. Furthmuller, *Phys. Rev. B* **1996**, *54*, 11169-11186.

[2] J. P. Perdew, K. Burke, M. Ernzerhof, *Phys. Rev. Lett.* **1996**, *77*, 3865-3868.

[3] P. E. Blochl, *Phys. Rev. B* **1994**, *50*, 17953-17979.
